# Bone regenerative effect of recombinant human bone morphogenetic protein-2 after cyst enucleation

**DOI:** 10.1186/s40902-016-0070-4

**Published:** 2016-05-26

**Authors:** Doo Yeon Hwang, Sung Woon On, Seung II Song

**Affiliations:** Department of Oral and Maxillofacial Surgery, Institute of Oral Health Science, Ajou University School of Medicine, 164, World Cup-ro, Yeongtong-gu, Suwon-si, Gyeonggi-do 16499 Republic of Korea

**Keywords:** rhBMP-2, Bone regeneration, Bone defect

## Abstract

**Background:**

The aim of this study is to quantitatively evaluate the effect of rhBMP-2 for repair of bone defects after cyst enucleation using the osteogenesis index (OI).

**Methods:**

Under general anesthesia, 10 patients (12 lesions) underwent oral or maxillofacial surgery for cyst enucleation. Postoperatively, 12 lesions were divided into two groups: group A (six lesions) was treated with absorbable collagen sponge (ACS) in combination with rhBMP-2, and group B (six lesions) was treated with ACS alone. After 3 months, cone-beam computed tomographic scans were obtained to measure changes in the volume of the lesions. We then calculated the OI of each group at two different Hounsfield units to determine any statistically significant difference between these two groups (Mann–Whitney *U* test).

**Results:**

As tested at the level of new bone, the mean OI was 72.37 % in group A and 55.08 % in group B —a statistically significant difference (*p* = 0.041). As tested at the level of mature bone, the mean OI was 27.47 % in group A and 18.88 % in group B, but the difference was not statistically significant (*p* = 0.394).

**Conclusions:**

The application of rhBMP-2 after maxillofacial cyst enucleation accelerated new bone formation in the bone defects. Thus, the use of rhBMP-2 in combination with ACS may be considered an alternative to conventional bone grafting in some patients with postoperative bone defects.

**Electronic supplementary material:**

The online version of this article (doi:10.1186/s40902-016-0070-4) contains supplementary material, which is available to authorized users.

## Background

Bone defects in the oral and maxillofacial region have many different causes, such as infection, trauma, lesions, or invasive surgery. Because such defects require faster healing and complete reconstruction to achieve functional and esthetic recovery, numerous studies and relentless efforts have been undertaken to fulfill these requirements. As a result, a variety of bone graft materials and osteogenic factors have been examined but have not led to ideal alternatives or substitutes, so relevant studies are ongoing.

Bone morphogenic proteins (BMPs) were discovered in 1965 by Urist [1], revealing their ability to differentiate undifferentiated osteogenic progenitor cells. Unlike conventional bone graft materials, which are only osteoconductive, BMPs are osteoinductive and therefore represent an optimal alternative to bone grafting for reconstruction of the oral and maxillofacial region [2]. Various applications of rhBMP-2 have been explored since it was approved in 2007 as a substitute for autografts in maxillary sinus augmentation and alveolar ridge defects around an extraction socket. Previous studies have demonstrated that rhBMP-2 is effective for cleft lip and palate, alveolar bone augmentation, sinus augmentation, osteonecrosis of the jaw, and reconstruction of the oral and maxillofacial region [3–5].

Intrabony cysts are common lesions of the oral and maxillofacial region and often require surgical removal. In many cases, after removal of the cyst, bone grafting is performed using a particle-type bone graft material. Recently, however, because bone graft materials may become dispersed or may migrate depending on the size or location of the defect and the bony housing of the lesion [6], absorbable collagen sponge (ACS) with absorbed BMPs has occasionally been used instead [7].

Although many studies have already shown the bone regenerative effects of rhBMP-2, most of these were in vitro or animal studies in which rhBMP-2 was added to other bone graft materials, and some of the clinical reports involved non-quantitative methods. Our study was intended to analyze the bone regenerative effects of rhBMP-2 quantitatively based on cone-beam computed tomographic (CBCT) images of patients treated with and without rhBMP-2.

## Methods

### Patients

We evaluated 38 patients who visited the Department of Oral and Maxillofacial Surgery at Ajou University Hospital in South Korea from January 1, 2014, through March 31, 2015, and who underwent cyst enucleation under general anesthesia. For our study, the following selection criteria were applied:

#### Inclusion criteria

- Both preoperative and 3-month postoperative CBCT images were available.
- ACS alone or rhBMP-2 plus ACS was used for bone defect repair.

#### Exclusion criteria

- Recipients of bone grafts (allogenic or xenogenic bone material).
- Patients with diseases related to bone metabolism (e.g., osteoporosis).

Based on these criteria, we selected 10 patients for study, two of whom each had one additional, independent lesion, for a total of 12 lesions. Human CBCT data were used in this study but patient consent was not necessary. This study was reviewed and approved by the institutional review board of Ajou University Hospital (AJIRB-MED-MDB-15-203).

### Methods

#### Study design

Two operators performed cyst enucleation for 12 lesions. After a retrospective chart review, we divided the 12 cases into two groups of six lesions each: group A was treated with rhBMP-2 plus ACS, and group B was treated with ACS alone. Preoperative and 3-month postoperative lesion volumes were measured on CBCT images, and the osteogenesis index was calculated in each case, after which, a statistical analysis was performed.

#### Surgical procedure

**Fig. 1:**
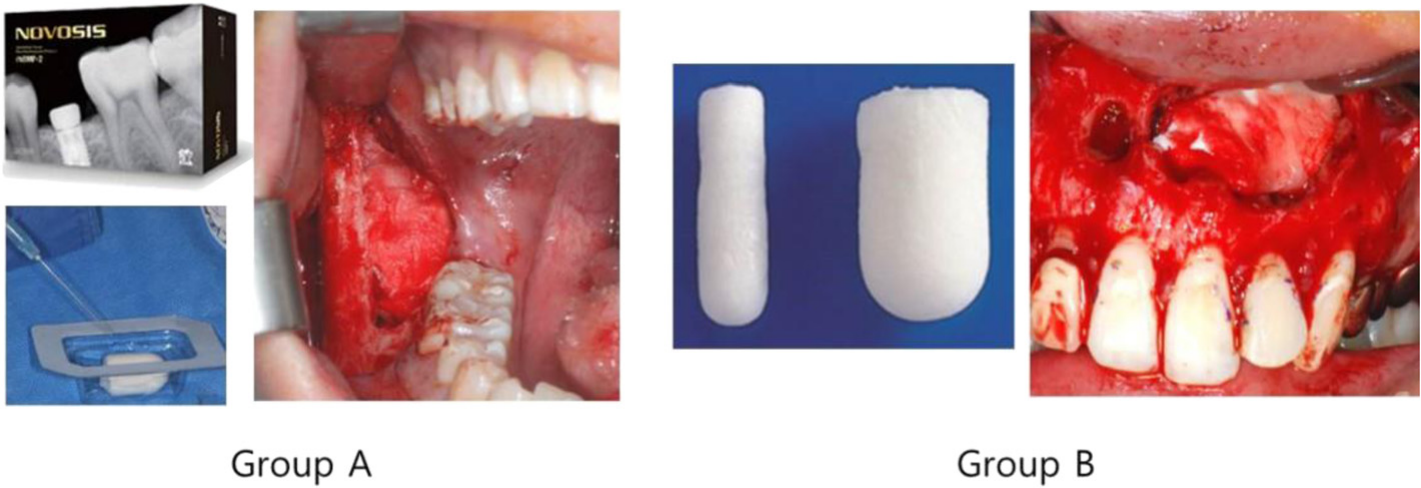
Surgical procedures for group A and group B. In group A, ACS with absorbed rhBMP-2 was applied to the bone defects. In group B, ACS alone was applied to the bone defects

After cyst enucleation under general anesthesia, ACS with absorbed rhBMP-2 was applied on the bone defects in group A and primary closure was completed. In group B, ACS alone was applied after cyst enucleation, with subsequent primary closure (Fig. 1). Up to five units of ACS was used and up to 1.0 mg of rhBMP-2 (1 mg/mL) was used, depending on the size of the lesion. The rhBMP-2 used in this study was NOVOSIS (Daewoong Pharmaceutical Company, Seoul, South Korea), and the ACS was either Ateloplug (Bioland, Cheonan, South Korea) or Rapiderm Plug (Dalim Tissen, Seoul, South Korea).

#### Lesion volume measurements

**Fig. 2:**
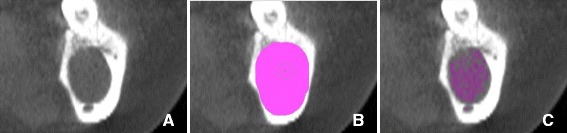
Selection of pixels within the range of Hounsfield units desired (selection process). **a** Original CBCT image. **b** Drawing of lesional area. **c** Selected pixels

**Fig. 3:**
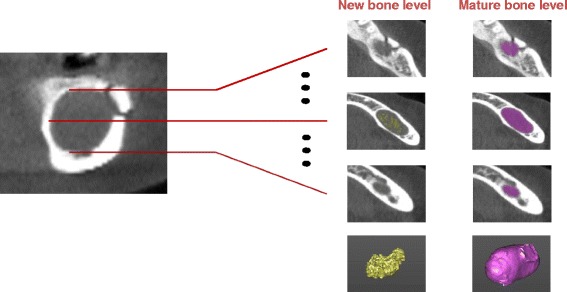
Remodeling to a three-dimensional model. Selection of pixels regarded as lesions at the new bone level and the mature bone level for all computed tomographic images

Each case was analyzed using Simplant software (Materialise Dental, Leuven, Belgium). From among the user’s tools, we selected only those pixels (Fig. 2) within the preset range of Hounsfield units (minimum = −1024 HU; maximum = 200 or 600 HU) for all the coronal or axial images believed to include lesions. The selected pixels were then remodeled into three-dimensional images, and the volumes were calculated. Hounsfield units ranged from −1024 (minimum) to 200 (new bone level) or 600 (mature bone level) (maximum), in reference to the studies performed by Norton and Gamble [8], Shapurian et al. [9], and Tajima et al. [10] (Fig. 3). Specifically, we defined two ranges: range 1 included −1024 < HU < 200 and range 2 included −1024 < HU < 600. For range 1, pixels with volumes of < 200 HU were regarded as a lesion; for range 2, pixels with volumes of < 600 HU were regarded as a lesion.

To minimize any possible visual errors during measurement, the patient’s head position on CBCT images was repositioned prior to volume measurement by using the line connecting the bilateral orbitales on the coronal plane, the line connecting the anterior nasal spine (ANS) and the cervical vertebra on the horizontal plane, and the line connecting the ANS to the posterior nasal spine (PNS) on the sagittal plane. To ensure accuracy, volume measurements were done twice in each case, and the mean results were used in this study.

#### Calculation of OI

**Fig. 4:**
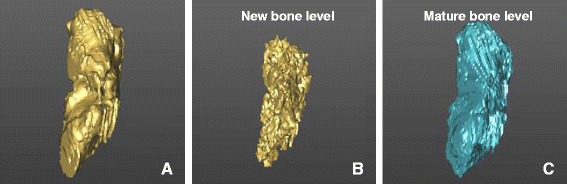
Initial lesion and reduced lesion after 3 months. **a** Preoperative lesion. **b** Postoperative lesion at new bone level. **c** Postoperative lesion at mature bone level

Preoperative lesion volume was defined as *V*_0_ and postoperative lesion volume as *V*_h_ (HU = 200 or 600) (Fig. 4). The OI was calculated as (*V*_0_ − *V*_h_)/*V*_0_ (%).

#### Assessment and statistical analysis

Using OI, we compared osteogenesis in groups A and B, and differences with a *p* value of < 0.05 were considered statistically significant. The Mann–Whitney *U* test and SPSS version 22.0 (IBM, NY, USA) were employed as the statistical technique and software program, respectively.

## Results

The patients ranged in age from 16 to 50 years (mean age = 36.25), and the ratio of male-to-female patients was 9:1. Of the 12 lesions, nine were located in the mandible and the other three in the maxilla. Histological findings for the individual lesions indicated that there were nine dentigerous cysts, two periapical cysts, and one keratocystic odontogenic tumor (KCOT) (Table 1). All the lesions healed with no remarkable complications.

**Table 1:** Characteristics of the 12 lesions

Group	Age (year)	Gender	Site	Diagnosis	*V*_0_ (cm^3^)
A (ACS + rhBMP-2)	16	M	Mandible	DC	14.82
	50	F	Mandible	DC	1.98
	21	M	Mandible	KCOT	6.47
	45	M	Mandible	DC	2.27
	41	M	Mandible	DC	3.14
	20	M	Mandible	DC	5.02
B (ACS alone)	51	M	Mandible	DC	2.30
	48	M	Maxilla	PC	6.64
	37	M	Mandible	DC	1.24
	37	M	Mandible	DC	2.67
	45	M	Maxilla	DC	2.95
	40	M	Mandible	PC	13.48

*DC* dentigerous cyst; *PC* periapical cyst; *KCOT* keratocystic odontogenic tumor

The measurements of preoperative lesion volume (*V*_0_) ranged from 1.24 to 14.82 cm^3^, with a mean of 5.25 cm^3^. The OI measurements at the level of new bone ranged from 29.88 to 88.21 %, with a mean of 63.72 %, and at the level of mature bone from 5.12 to 55.33 %, with a mean of 23.17 %. When postoperative lesion volume was measured, increases in the number of Hounsfield units were seen throughout the lesions, especially around the margins.

**Fig. 5:**
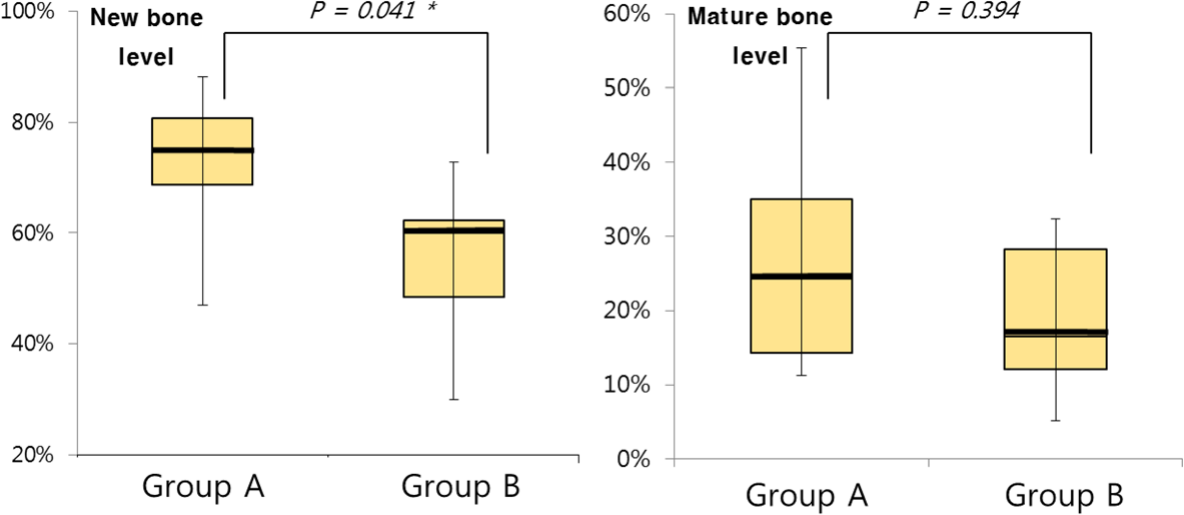
OI values for group A and group B. The OI of groups A and B at the level of new bone (*left*) and the level of mature bone (*right*). At the new bone level, a statistically significant difference was seen between group a and group b (*p* < 0.05)

When tested at the new bone level, the mean OI was 72.37 % for group A and 55.08 % for group B—a statistically significant difference (*p* = 0.041). However, when tested at the mature bone level, the mean OI was 27.47 % for group A and 18.88 % for group B, a difference that did not reach statistical significance (*p* = 0.394) (Table 2 and Fig. 5).

**Table 2:** Preoperative and 3-month postoperative mean volume (±SD) and mean OI (±SD) of lesion

Group	*V*_0_	New bone		Mature bone	
		*V*_200_ (cm^3^)	OI (%)	*V*_600_ (cm^3^)	OI (%)
A (ACS **+** rhBMP-2)	5.62 ± 4.82	1.67 ± 1.60	72.37 ± 14.39	4.56 ± 4.57	27.47 ± 17.09
B (ACS alone)	4.88 ± 4.60	1.79 ± 1.14	55.08 ± 15.26	3.81 ± 3.21	18.88 ± 11.15

*V*_*0*_ preoperative volume; *V*_*200*_ volume at new bone level (< 200 HU); *V*_*600*_ volume at mature bone level (< 600 HU); *OI* osteogenesis index

## Discussion

Human bone regenerates through patterns of maturation similar to those of bone growth in response to bone defects of any cause. Stable bone healing is achieved when there is an adequate blood supply and immobilization at the site of the defect. For the first 4 weeks, angiogenic and osteogenic cells originate from the surrounding bone walls and periosteum, while woven bone forms around the defect. These processes are governed by various cytokines and growth factors [11–15].

Ettl et al. suggested that although primary closure after cyst enucleation can be accomplished without bone grafts, further research regarding growth factors, osteoblasts, stem cells, and other components is needed to understand this process more fully [16]. Bone defects up to 3 cm in diameter usually undergo complete ossification after 12 months, while larger bone defects may require a longer period of ossification (24 months or more) [17, 18]. In spite of the obvious need for additional treatment to accelerate healing (e.g., bone grafting), such measures cannot always be taken when possible complications such as infection or migration are of concern. Recently, ACS with absorbed rhBMP-2 has been applied in such situations.

In his primate study, Boyne reported that rhBMP-2 alone was useful even without bone graft material for the reconstruction of facial bone defects after mandibular hemisection, implant, and cleft repair [19]. After reviewing the literature on alveolar ridge augmentation, maxillary sinus augmentation, and/or extraction socket preservation, Freitas et al. reported that ACS with absorbed rhBMP-2 appeared to function as an alternative to autografting in alveolar ridge or maxillary sinus augmentation [20]. Balaji reported the use of rib grafting and rhBMP-2 following removal of an aneurysmal bone cyst [2], and in 2014, Lee et al. also reported the use of rhBMP-2 and β-TCP/HA (tricalcium phosphate/hydroxyapatite) in five patients with cysts [21].

Unfortunately, however, there have been some limitations to the use of rhBMP-2 despite the successful outcomes described above. These include the shorter half-life of BMP-2 and its rapid elimination at the application site, which requires a high dose of BMP-2 and thus expensive medical costs, overgrowth of bone, and unwanted side effects, including swelling due to immune reactions [7, 22, 23]. According to a recent report, excessively high doses of BMP-2 may cause oral squamous cell carcinoma [24]. However, we did not observe complications in any of the patients treated at our hospital.

One can compensate for the abovementioned disadvantages of BMP by selecting an appropriate carrier. Currently available carriers include HA, TCP, DBM, hydrogel, and ACS. Referring to the existing literature, Geiger et al. described “enhancement of osteogenic activity of BMP with a restrictive release of BMP at an effective dose during a period coincident with the accumulation and proliferation of target cells” [25]. Li and Wozney reported that the releasing periods of rhBMP-2 were at least twice as long when treatment included the ACS compared with the control treatment without the sponge, and ACS is an appropriate carrier for BMP application [26]. In contrast, in 2008, Carter et al. mentioned that although ACS is of value for the delivery of BMP and offers good space-maintaining ability, it should be used with caution because its overcompressed use may interfere with normal bone formation [7].

Bone density can be assessed by measuring Hounsfield units and has different values depending on the type of bone. Very dense cortical bone is expressed as 600 HU or more, the dense cortical/spongy bone as 400 through 600 HU, and low-density bone as 200 HU or less [8, 9]. In 2013, Tajima et al. reported that the density of peri-implant, new bonelike tissue ranged from 185 to 713 HU (mean ± SD = 323 ± 156.2) [10].

Huh et al. found that combination therapy with bovine bone (Bio-Oss) and rhBMP-2 leads to more new bone generation than does bovine bone monotherapy and that rhBMP-2 enhanced bone regeneration [27]. In our study, the mean OI was higher in the rhBMP-2 treatment group A than in the group B, and the difference was statistically significant for new bone levels with maximum number of Hounsfield units set at 200. This result suggests that rhBMP-2 contributes significantly to new bone generation in the human body as well.

This study had the following limitations: difficulty in determining the margin when measuring postoperative lesion volume owing to the need for intraoperative osteotomy to approach the lesion; several diagnoses of the lesions; preoperative secondary infections due to the lesions; the degree of defect in the bony housing; and no consideration of the number of absorbable collagen sponges or the quantity of rhBMP-2 actually applied during the operation. Nevertheless, this study is meaningful in that we used a quantitative method to analyze the effect of rhBMP-2 in human subjects. Further studies will be needed to perform histomorphometric analyses of the effects of rhBMP-2 in the human body.

## Conclusions

When rhBMP-2 was used to repair bone defects that remain after cyst enucleation, new bone formation was increased. Thus, the combination of rhBMP-2 and ACS could be considered as an alternative to conventional bone grafts. We believe that rhBMP-2 is worthy of being applied to bone defects in the oral and maxillofacial region in certain cases.

### Ethics approval and consent

Human CBCT data were used in this study, but consent for patients was not necessary. This study was reviewed and approved by the Institutional Review Board of Ajou University Hospital (AJIRB-MED-MDB-15-203).

### Availability of data and materials

The dataset supporting the conclusions of this article is included in Additional file 1.

## Additional file

Additional file 1: Case form and result of data. (XLSX 13 kb)

## Abbreviations

ACS: absorbable collagen sponge
OI: osteogenesis index
